# A study of genes encoding cytokines (*IL6*, *IL10*, *TNF*), cytokine receptors (*IL6R*, *IL6ST*), and glucocorticoid receptor (*NR3C1*) and susceptibility to bronchopulmonary dysplasia

**DOI:** 10.1186/s12881-014-0120-7

**Published:** 2014-11-01

**Authors:** Johanna M Huusko, Minna K Karjalainen, Mari Mahlman, Ritva Haataja, M Anneli Kari, Sture Andersson, Gergely Toldi, Outi Tammela, Mika Rämet, Pascal M Lavoie, Mikko Hallman, on behalf of Gen-BPD Study Group

**Affiliations:** Department of Pediatrics, Institute of Clinical Medicine, Medical Research Center Oulu, Oulu University Hospital and University of Oulu, Oulu, Finland; Department of Children and Adolescents, Oulu University Hospital, Oulu, Finland; Biocenter Oulu, Oulu, Finland; Children’s Hospital, Helsinki University Central Hospital and University of Helsinki, Helsinki, Finland; First Department of Pediatrics, Semmelweis University, Budapest, Hungary; Department of Pediatrics, Tampere University Hospital, Tampere, Finland; BioMediTech, University of Tampere, Tampere, Finland; Child and Family Research Institute of British Columbia, Vancouver, Canada

**Keywords:** Bronchopulmonary dysplasia, Epistasis, Glucocorticoid receptor, Interleukin, Preterm infant, Single nucleotide polymorphism

## Abstract

**Background:**

Bronchopulmonary dysplasia (BPD) is a common chronic lung disease associated with very preterm birth. The major risk factors include lung inflammation and lung immaturity. In addition, genetic factors play an important role in susceptibility to moderate-to-severe BPD. In this study, the aim was to investigate whether common polymorphisms of specific genes that are involved in inflammation or differentiation of the lung have influence on BPD susceptibility.

**Methods:**

Genes encoding interleukin-6 (*IL6*) and its receptors (*IL6R* and *IL6ST*), IL-10 (*IL10*), tumor necrosis factor (*TNF*), and glucocorticoid receptor (*NR3C1*) were assessed for associations with moderate-to-severe BPD susceptibility. Five *IL6*, nine *IL6R*, four *IL6ST*, one *IL10*, two *TNF*, and 23 *NR3C1* single nucleotide polymorphisms (SNPs) were analyzed in very preterm infants born in northern Finland (56 cases and 197 controls) and Canada (58 cases and 68 controls). IL-6, TNF and gp130 contents in umbilical cord blood, collected from very preterm infants, were studied for associations with the polymorphisms. Epistasis (i.e., interactions between SNPs in BPD susceptibility) was also examined. SNPs showing suggestive associations were analyzed in additional replication populations from Finland (39 cases and 188 controls) and Hungary (29 cases and 40 controls).

**Results:**

None of the studied SNPs were associated with BPD nor were the *IL6*, *TNF* or *IL6ST* SNPs associated with cord blood IL-6, TNF and gp130, respectively. However, epistasis analysis suggested that SNPs in *IL6ST* and *IL10* were associated interactively with risk of BPD in the northern Finnish population; however, this finding did not remain significant after correction for multiple testing and the finding was not replicated in the other populations.

**Conclusions:**

We conclude that the analyzed SNPs within *IL6*, *IL6R*, *IL6ST*, *IL10*, *TNF*, and *NR3C1* were not associated with BPD. Furthermore, there was no evidence that the studied SNPs directly contribute to the cord blood protein contents.

**Electronic supplementary material:**

The online version of this article (doi:10.1186/s12881-014-0120-7) contains supplementary material, which is available to authorized users.

## Background

Bronchopulmonary dysplasia (BPD) is a chronic lung disease that affects infants born very preterm. Despite advances in treatment practices (including antenatal glucocorticoid treatment), BPD continues to be a major cause of neonatal morbidity and mortality in these infants. Furthermore, infants with BPD are at increased risk of respiratory morbidities in later life, and lung function defects may persist even into adulthood [1]. Major risk factors that predispose infants to BPD include prematurity [2], fetal growth restriction [3], and lung inflammation [4]. In addition to environmental stressors, the results of twin studies indicate high heritability for BPD and that the genetic factors have an important role in predisposition to BPD [5-7] accounting for up to 79% of the variance in liability to moderate-to-severe BPD [6]. Despite numerous candidate gene studies and two genome-wide association studies, the genetic background of BPD remains incompletely characterized [8-11].

Infants with BPD often have a simplified lung structure. It has been proposed that initiation of an inflammatory cascade in immature lung tissue interferes with the normal course of septation and alveolar development. This leads to fewer, simplified, and larger alveoli and dysmorphic pulmonary vasculature, which are typical features of impaired alveolarization and common findings in BPD [4,12]. In addition, the inflammatory cytokines are strongly associated with the airway and interstitial lung injury, and thus, several inflammatory mediators have been studied for associations with BPD [13]. Previously, elevated levels of cytokines were observed in blood, cord blood or amniotic fluid from infants who subsequently developed BPD [14-16]. In particular, elevated levels of proinflammatory cytokine interleukin 6 (IL-6) in blood after birth [14], glycoprotein 130 (gp130, also known as IL-6 signal transducer) in cord blood [15] and reduced expression of IL-10 in placenta [17] were associated with risk of BPD.

This case–control study was performed to assess whether genes involved in inflammation and lung maturation are associated with susceptibility to moderate-to-severe BPD, defined by the need of supplemental oxygen at the corrected age of 36 weeks. The genes encoding IL-6 (*IL6*), its receptors IL-6R (*IL6R*) and gp130 (*IL6ST*), IL-10 (*IL10*), tumor necrosis factor (*TNF*) formerly known as TNF-alpha, were studied. In addition, the gene encoding glucocorticoid receptor (*NR3C1*) was selected. Glucocorticoids have strong anti-inflammatory effects, and they additionally influence growth and differentiation. The actions of both endogenous and synthetic glucocorticoids are mediated by the glucocorticoid receptor [18,19]. Furthermore, associations between specific polymorphisms and cord blood serum IL-6, TNF and gp130 contents were studied. Finally, epistasis was investigated; i.e., whether interactions between polymorphisms in the six analyzed genes affect disease susceptibility.

## Methods

Written informed consent was obtained from the parents, and the study was approved by the Ethics Committee of Oulu University Hospital, the University of British Columbia Clinical Research Ethics Board, the University of Alberta Ethics Board, and the Semmelweis University Hospital Ethical Committee.

### Diagnosis of BPD and infant inclusion criteria

The diagnosis of BPD was based on a requirement for supplemental oxygen or ventilation at 36 wk post menstrual age (PMA). For infants who required supplemental oxygen for a minimum of 28 d, the severity of BPD was graded (according to National Institute of Child Health and Human Development criteria) as mild BPD (no supplemental O_2_ requirement or ventilation at 36 wk PMA), moderate BPD (supplemental O_2_ requirement <30% at 36 wk PMA), or severe BPD (supplemental O_2_ requirement ≥30% or ventilation at 36 wk PMA) [20]. The oxygen reduction test [21] was performed for infants born in Finland after 2009; the test eliminated less than 10% of all cases of moderate BPD. Although infants from different centers were included in the study, the possible differences in BPD diagnosis were small since the transcutaneous oxygen saturation limits were kept within a close range (lower limit from 86 to 90%; upper limit from 92 to 95%), the oxygen saturation test was similar in each center, and of all infants studied only one third of the Canadian population was treated in moderately high altitude (~670 m).

In case–control analyses, infants with no-to-mild BPD were controls and infants with moderate-to-severe BPD were cases. This approach was based on twin studies that demonstrated significant heritability in moderate-to-severe BPD but less in mild BPD [6]. We excluded infants with malformations and those who died before the diagnosis. Additionally, only one infant was included from each monozygotic twin pair.

### Study populations

All infants were born at gestational age (GA) <31 wk. Clinical characteristics are presented in Table 1. The northern Finnish population (n =253) was prospectively recruited at Oulu University Hospital in 1997–2010. Only infants with parents of Finnish origin were included. The study population that originated Canada (n =126) comprised infants born in neonatal intensive care units in Vancouver and Edmonton in 2006–2008, as described previously [22]. These infants were of European descent. In addition, two replication populations were included. The first population comprised infants born in Finland (n =227) in the university hospitals of Oulu, Kuopio, Tampere, and Helsinki in 2010–2012 and in the University Hospital of Turku in 2001–2006 and 2011–2012. This population was not included in the original analyses because samples were prospectively collected during or after the original analyses. The second replication population (n =69) included infants of European descent born in Semmelweis University Hospital, Budapest, Hungary in 1995–2002.

**Table 1:** **Clinical characteristics of the northern Finnish, Canadian, replication Finnish, and replication Hungarian preterm cohorts**

	**Northern Finnish**		**Canadian**		**Replication Finnish**		**Replication Hungarian**	
	**BPD cases**	**Controls**	**BPD cases**	**Controls**	**BPD cases**	**Controls**	**BPD cases**	**Controls**
**n**	**56**	**197**	**58**	**68**	**39**	**188**	**29**	**40**
Gestational age, weeks^1^	27.5 ± 1.9 (24–30)	28.7 ± 1.7 (23–30)***	25.9 ± 1.6 (23–30)	27.6 ± 1.3 (24–30)***	26.5 ± 1.9 (24–30)	27.5 ± 2.0 (23–30)**	26.6 ± 1.8 (24–30)	28.6 ± 1.4 x 24–30)***
Gestational age <28 wk, n (%)	29 (51.8)	64 (32.5)**	48 (82.8)	32 (47.1)***	30 (76.9)	88 (46.8)**	19 (65.5)	6 (15.0)***
Birth weight, grams^1^	938 ± 268 (495–1555)	1184 ± 313 (370–1850)***	834 ± 221 (385–1420)	1162 ± 243 (630–1900)***	878 ± 257 (440–1470)	1074 ± 308 (520–1970)***	928 ± 226 (510–1500)	1179 ± 193 (650–1500)***
Birth weight *Z*-score^1,2^	−1.4 ± 1.41 (−4.5–1.8)	−0.9 ± 1.22 (−5.5–1.7)*	NA	NA	−1.4 ± 1.38 (−4.0–1.1)	−0.9 ± 1.3 (−4.0–2.6)	NA	NA
IUGR ≤ −2 SD, n (%)	21 (37.5)	33 (16.8)**	11 (19.0)	3 (4.4)*	13 (33.3)	41 (21.8)	NA	NA
Male gender, n (%)	34 (60.7)	111 (56.3)	29 (50.0)	30 (44.1)	26 (66.7)	94 (50.0)	16 (55.2)	24 (60.0)
Singletons, n (%)	47 (83.9)	164 (83.2)	39 (67.2)	44 (64.7)	29 (74.4)	138 (73.4)	26 (89.7)	33 (82.5)
Severe BPD, n (%)	21 (37.5)		21 (36.2)		18 (46.2)		NA	

*P <0.05, **P <0.01, ***P <0.001; Mann–Whitney *U*-test for continuous variables, *X* ^*2*^-test for dichotomous variables; ^1^mean ± standard deviation (range); ^2^Birth weight *Z*-score describing fetal intrauterine growth. BPD cases were infants with moderate-to-severe BPD, and controls were infants with no-to-mild BPD. BPD, bronchopulmonary dysplasia; IUGR, intrauterine growth restriction; NA, information not available.

### DNA sample preparation

For the samples collected in Finland, genomic DNA was extracted from buccal cells, umbilical cord blood, and blood spots dried on paper with Chelex 100 (Bio-Rad, Hercules, CA, USA), UltraClean DNA blood Isolation kit (MO BIO Laboratories Inc., Carlsbad, CA, USA), and MO BIO UltraClean BloodSpin DNA Isolation Kit (MO BIO Laboratories), respectively. For buccal cell and paper blood DNA samples, whole-genome amplification was performed, as described previously [23]. For samples that originated in Canada, genomic DNA was extracted from umbilical cord tissue and cell samples, whole blood specimens, and tracheal aspirate samples with the ChargeSwitch gDNA Mini Tissue Kit (Invitrogen, Carlsbad, CA, USA), QIAamp DNA Blood Midi Kit (Qiagen, Hilden, Germany), and ChargeSwitch gDNA Buccal Cell Kit (Invitrogen), respectively. DNA from the Hungarian samples was extracted from dried whole blood spots with saponin and Chelex-100, as described previously [24].

### SNP selection and genotyping

Tagging SNPs (tSNPs) that capture most of the common genetic variation in the selected regions based on linkage disequilibrium (LD) were selected by using HapMap data (release 24/phases I&II) [25] for the CEU population (CEPH; Utah residents with ancestry from northern and western Europe). A minor allele frequency (MAF) cut-off value of 0.1 and an r^2^ cut-off value of 0.9 were used for pairwise tagging. A total of 44 SNPs (five SNPs in *IL6*, nine in *IL6R*, four in *IL6ST*, one in *IL10*, two in *TNF*, and 23 in *NR3C1*) were selected for genotyping. The analyzed SNPs are listed in Tables 2 and 3.

**Table 2:** ***IL6*** **,** ***IL6R*** **,** ***IL6ST*** **,** ***IL10*** **, and** ***TNF*** **minor allele frequencies in cases and controls in the northern Finnish and Canadian populations**

		**Northern Finland, n =253**		**Canada, n =126**	
**Gene**	**Polymorphism** ^**1**^	**Case, control minor allele frequency**	***P*** **value**	**Case, control minor allele frequency**	***P*** **value**
*IL6*	rs2069827	0.286, 0.265	0.6603	0.129, 0.119	0.8126
*IL6*	rs1800797	0.438, 0.454	0.7592	0.379, 0.388	0.8872
*IL6*	rs1800795	0.438, 0.454	0.7523	0.404, 0.404	0.9884
*IL6*	rs2069832	0.427, 0.457	0.5814	0.397, 0.404	0.8990
*IL6*	rs2069840	0.222, 0.237	0.7464	0.276, 0.243	0.5480
*IL6R*	rs4845617	0.400, 0.426	0.6304	0.276, 0.390	0.0568
*IL6R*	rs1386821	0.214, 0.203	0.795	0.172, 0.213	0.4144
*IL6R*	rs4075015	0.444, 0.423	0.6859	0.509, 0.462	0.4614
*IL6R*	rs4601580	0.417, 0.460	0.4527	0.491, 0.500	0.8898
*IL6R*	rs4553185	0.368, 0.434	0.2218	0.474, 0.463	0.8627
*IL6R*	rs4453032	0.273, 0.320	0.3479	0.431, 0.449	0.7804
*IL6R*	rs4845374	0.116, 0.121	0.8845	0.103, 0.096	0.8352
*IL6R*	rs4240872	0.389, 0.386	0.9567	0.293, 0.257	0.5259
*IL6R*	rs4072391	0.343, 0.361	0.7265	0.216, 0.184	0.5295
*IL6ST*	rs10471960	0.080, 0.104	0.4582	0.129, 0.125	0.9184
*IL6ST*	rs10940495	0.259, 0.303	0.3717	0.267, 0.239	0.6055
*IL6ST*	rs11739048	0.089, 0.105	0.6247	0.155, 0.140	0.7295
*IL6ST*	rs6875155	0.071, 0.103	0.3233	0.121, 0.125	0.9173
*IL10*	rs3024493	0.120, 0.153	0.3908	0.121, 0.191	0.1270
*TNF*	rs1799964	0.136, 0.155	0.6362	0.198, 0.169	0.5503
*TNF*	rs1800629	0.100, 0.144	0.2359	0.190, 0.132	0.2147

^1^SNP accession numbers in dbSNP database (http://www.ncbi.nlm.nih.gov/SNP).

**Table 3:** ***NR3C1*** **minor allele frequencies in cases and controls in the northern Finnish and Canadian populations**

	**Northern Finland, n =253**		**Canada, n =126**	
***NR3C1*** **polymorphism** ^**1**^	**Case, control minor allele frequency**	***P*** **value**	**Case, control minor allele frequency**	***P*** **value**
rs17287758	0.107, 0.107	0.9869	0.155, 0.154	0.9867
rs17209237	0.264, 0.237	0.5685	0.219, 0.235	0.7640
rs6196	0.200, 0.184	0.7034	0.198, 0.184	0.7709
rs10482689	0.107, 0.105	0.9381	0.155, 0.154	0.9867
rs10482682	0.339, 0.319	0.6811	0.327, 0.347	0.7529
rs6188	0.309, 0.296	0.7897	0.362, 0.353	0.8802
rs10482672	0.128, 0.132	0.9191	0.172, 0.125	0.2892
rs33383	0.460, 0.500	0.4799	0.482, 0.441	0.5248
rs2918417	0.304, 0.293	0.825	0.366, 0.353	0.8301
rs6877893	0.464, 0.482	0.7372	0.466, 0.441	0.6988
rs4912905	0.241, 0.241	0.9992	0.241, 0.213	0.5945
rs2918415	0.196, 0.178	0.6609	0.198, 0.184	0.7709
rs17399352	0.236, 0.218	0.6763	0.172, 0.206	0.5001
rs2963155	0.321, 0.321	0.9854	0.319, 0.272	0.4151
rs9324924	0.373, 0.409	0.4954	0.397, 0.346	0.4034
rs7701443	0.366, 0.359	0.8903	0.353, 0.353	0.9933
rs4244032	0.134, 0.153	0.6201	0.190, 0.169	0.6714
rs4607376	0.500, 0.495	0.9247	0.500, 0.449	0.4146
rs13182800	0.116, 0.147	0.4074	0.190, 0.169	0.6714
rs12054797	0.255, 0.320	0.1888	0.302, 0.294	0.8953
rs4912910	0.382, 0.414	0.5472	0.353, 0.360	0.9100
rs12656106	0.446, 0.407	0.4619	0.431, 0.508	0.2274
rs12655166	0.268, 0.324	0.2641	0.302, 0.294	0.8953

^1^SNP accession numbers in dbSNP database (http://www.ncbi.nlm.nih.gov/SNP).

Genotyping was performed with the Sequenom iPLEX Gold assay. Three SNPs deviated from Hardy–Weinberg equilibrium (HWE): *IL6R* rs4075015 and rs4453032 in the northern Finnish population and *IL6* rs2069840 in the Canadian population. Because these SNPs deviated from HWE in only one of the populations, they were included in analyses. In the replication study, *IL6ST* rs10471960 and *IL10* rs3024493 SNPs were genotyped by PCR-RFLP analysis with primer pairs of 5′-CAGAGTGGCTTAGGGACAGTT-3′ (forward) and 5′-ACTCGCAGCATCACTACCAAT-3′ (reverse) for rs10471960 and 5′-GGGTGGCTGCTAGGCATTT-3′ (forward) and 5′-GAATAGCCCCCTTGTCCCTTC-3′ (reverse) for rs3024493, with restriction enzymes *BssSI* and *BamHI* (New England Biolabs, Ipswich, MA, USA), respectively.

### Analysis of IL-6, TNF and gp130 in umbilical cord blood specimens

IL-6, TNF and gp130 protein contents were measured from umbilical cord blood specimens collected from a cohort of very preterm infants born in Oulu University Hospital during 1998–2002, as described previously [15]. A total of 120 infants were included in the analysis: 35 infants subsequently developed moderate-to-severe BPD and 85 infants had no-to-mild BPD. The protocol of the antibody-based microarray has been previously described in detail [26]. The protein content of blood specimens are reported as fluorescence units (FUs). Additionally, the association between cord blood proteins IL-6, TNF and gp130 and SNPs of the encoding genes was studied; including 100 infants with DNA sample available.

### Statistical analyses

Case–control comparisons for the continuous and dichotomous clinical characteristics (GA, birth weight, intrauterine growth expressed as *Z*-score, intrauterine growth restriction [IUGR] ≤ −2 SD, gender, and proportion of singletons) as well as the cord blood IL-6, TNF and soluble gp130 contents were performed by the nonparametric Mann–Whitney *U*-test and the Pearson *X*^*2*^ test with SPSS Statistics 20.0 (IBM Corporation, Armonk, New York, USA). Differences in the cord blood IL-6, TNF and gp130 contents among the genotypes were analyzed with Mann–Whitney *U*-test and Kruskal–Wallis test. Haploview version 4.2 [27] was used for comparing case–control allele and haplotype frequencies (*X*^*2*^ tests), testing for HWE, and obtaining pairwise LD values (D′ and r^2^, where D′ refers to the strength of LD and r^2^ describes the correlation coefficient between the two loci; values close to 1 refer to strong LD or correlation between the two SNPs, respectively). PLINK 1.07 [28] was used for logistic regression analyses (to take the effect of potential risk factors into account together with the genetic factors).

Because some of the 44 SNPs included in the study were in LD with each other and thus not considered independent markers, SNPSpD [29], a method that takes LD between SNPs into account, was used to calculate the effective number of independent SNPs for each of the six genes. This resulted in a total of 32 independent SNPs; using the Bonferroni correction for multiple testing, a *P* value of <0.0016 was considered significant.

Pairwise SNP–SNP interaction analyses were performed with the *epistasis* option in PLINK. The software uses logistic regression (for dichotomous phenotype) to provide an odds ratio (OR) for the interaction of each pair of SNPs by considering pairwise combinations of all of the SNPs. Redundant SNPs were excluded from the epistasis analysis based on the LD measurements (strong LD): only one SNP from each haploblock generated by Haploview was included (see Additional file 1). Additionally, SNPs were excluded if they showed significant deviation from HWE. With these exclusion criteria, 22 SNPs were included in the analysis, resulting in 231 pairwise SNP–SNP comparisons. The multiple testing–corrected significance threshold was *P* <0.00022.

### Genetic power of the study

The power of the study was estimated by the Genetic Power Calculator [30] with an additive risk model (the allelic 1 degree of freedom test assuming a causal SNP with a MAF range of 0.1–0.5 and a BPD prevalence of 0.2). Our population of very preterm infants, which included 114 cases and 265 controls (the combined northern Finnish and Canadian population) provided an estimate of 80% power (alpha =0.05) to detect genotypic relative risks of 1.56–1.7 for risk-allele carrier heterozygotes.

## Results

### Clinical characteristics of the BPD and control infants

Some of the clinical characteristics, such as GA, birth weight, and birth weight *Z*-score (birth weight adjusted for gestational age), differed significantly between infants with moderate-to-severe BPD and control infants with no-to-mild BPD (Table 1). There were no differences between cases and controls in the number of fetuses per pregnancy or in gender distribution (Table 1). Differences in the clinical characteristics were taken into account in the analyses when applicable.

### *IL6*, *IL6R*, *IL6ST*, *IL10*, *TNF* and *NR3C1* polymorphisms and haplotypes in BPD cases and controls

The allele frequency distribution of the 44 SNPs in BPD cases and controls was studied. None of the SNPs were associated with BPD in the northern Finnish or Canadian populations (Tables 2 and 3), nor did the clinical risk factors affect the results when included in the logistic regression analyses. When the northern Finnish and Canadian populations were combined, there were no significant associations with BPD (data not shown). There were no statistically significant haplotype associations with BPD in the genes studied when the two populations were analyzed separately or combined (data not shown).

### Analysis of IL-6, TNF and gp130 proteins in cord blood

Cord blood IL-6, TNF and gp130 were higher in very preterm infants who subsequently developed BPD (n =35) than in those who did not develop BPD (n =85) (median 1,299 FU in cases vs. 1,054 FU in controls, *P* =0.34; 514 FU in cases vs. 347 FU in controls, *P* =0.002; 46,419 FU in cases vs. 34,063 FU in controls, *P* <0.001, respectively). Higher gp130 content predicted the risk of moderate-to-severe BPD as reported previously [15]. There were no statistically significant differences in the measures of cord blood IL-6, TNF or gp130 among the genotypes of the *IL6*, *TNF* or *IL6ST* SNPs, respectively. Small number of cases or individuals with the minor alleles of the SNPs may have limited the power for statistical significance.

### Study of epistasis (SNP–SNP interactions)

Pairwise SNP–SNP interaction analyses were performed to identify epistasis between genes located in different genomic regions or chromosomes and to assess the interactions for associations with BPD susceptibility. A total of 231 valid SNP–SNP tests were performed with the 22 SNPs included in the analyses (corrected *P* value threshold <0.00022). In the northern Finnish population, there was a SNP–SNP interaction that showed borderline significance for risk of moderate-to-severe BPD; this interaction was between *IL6ST* rs10471960 and *IL10* rs3024493 SNPs (*P* =0.0003, OR_interaction_ =35.4; Table 4). The homozygote carriers of major alleles of both SNPs (i.e., carrying both AA and GG genotypes of *IL6ST* rs10471960 and *IL10* rs3024493, respectively) were at higher risk of BPD. Furthermore, the cases tended to have a higher combined major allele (A-G) frequency compared to controls (83.8% vs. 75.3%, respectively). Thus, a combination of the major alleles could increase susceptibility to BPD or the minor alleles could be protective. This interaction between *IL6ST* rs10471960 and *IL10* rs3024493 SNPs was not detected in the Canadian population (*P* =0.65) or when the populations were combined (*P* =0.020). In addition, no statistically significant interactions were detected in the Canadian population (*P* >0.005) or in the combined northern Finnish and Canadian populations (*P* >0.01).

**Table 4:** **Results of epistasis analysis in the northern Finnish population**

**Chr 1**	**Gene 1**	**SNP 1**	**Chr 2**	**Gene 2**	**SNP 2**	***P*** value of interaction*
1	*IL10*	rs3024493	5	*IL6ST*	rs10471960	0.0003
1	*IL6R*	rs1386821	7	*IL6*	rs2069832	0.0025
5	*NR3CI*	rs17209237	6	*TNF*	rs1800629	0.0049
1	*IL10*	rs3024493	5	*NR3C1*	rs17209237	0.0063

*None of the interactions were significant after correcting for multiple comparisons (*P* <0.0002 as threshold for significance). Only SNP x SNP interactions with *P* values <0.01 are shown.

### Replication study of *IL6ST* rs10471960 and *IL10* rs3024493 SNPs in BPD susceptibility

Due to the suggestive signal in the epistasis analysis in the northern Finnish population, the *IL6ST* rs10471960 and *IL10* rs3024493 SNPs were further analyzed in the replication population that included the additional Finnish and Hungarian populations. In the single marker association analysis, these two SNPs were not associated with BPD in the replication Finnish or Hungarian populations, or when the replication populations were combined with the initial populations. In the epistasis analyses, the interaction between the two SNPs was not significant in the replication Finnish (*P* =0.30) or Hungarian (*P* =0.61) populations, or when the initial northern Finnish and Canadian populations were combined with the replication populations (*P* =0.13). The frequencies of the combined major alleles remained similar to those observed in the northern Finnish population (83.8% in cases vs. 75.3% in controls), but the difference in the frequency between cases and controls was smaller in the sample set that included all of the studied populations (76.1% in cases vs. 73.8% in controls).

## Discussion

Despite the remarkable advances in perinatal care, BPD continues to be a major complication of prematurity. The increased survival of extremely preterm infants has contributed to an overall increase in the incidence of BPD with a long-term risk of respiratory dysfunction [1,2]. BPD occurs as the result of complex gene–environment interactions, but the etiology of BPD remains incompletely understood. Identification of the genetic component by family studies has highlighted the importance of genetic factors in predisposition to BPD [9]. Several polymorphisms have been associated with BPD in candidate gene and genome-wide association studies, but the associations have not been replicated at a statistically significant level [8,10,11]. However, considering the high (~80%) heritability of BPD, only a small part of this heritability can be explained by the potential susceptibility SNPs discovered thus far.

In the present case–control study, we investigated whether the *IL6*, *IL6R*, *IL6ST*, *IL10*, *TNF*, and *NR3C1* genes were associated with susceptibility to BPD or whether the genes have effect on cord blood serum protein levels at the time of birth. These genes were selected based on their involvement in inflammatory responses and lung maturation. To our knowledge, no previous genetic studies of BPD have reported significant associations between any of these genes and moderate-to-severe BPD. Previous studies of *TNF* polymorphisms yielded inconsistent results or associations that have not been replicated in subsequent studies [31-33]. In addition to single-SNP analyses, we investigated whether there is evidence for epistasis between these genes in predisposition to BPD. None of the 44 polymorphisms that we studied showed significant associations with BPD in the single marker case–control analyses. There were no significant associations between cord blood IL-6, TNF and gp130 and polymorphisms of the encoding genes. In the epistasis study, a suggestive interaction between *IL6ST* rs10471960 and *IL10* rs3024493 SNPs was observed in the population that originated in the northern Finland (*P* =0.0003). This interaction did not remain significant for predisposition to moderate-to-severe BPD after correction for multiple comparisons, and the detected interaction was not replicated in the additional studied populations.

Epistasis may be one of the factors that account for the missing heritability in complex diseases [34]. However, assessing whether the detected interactions in epistasis analyses represent true gene–gene interactions is not a straightforward task. To our knowledge, interactions between *IL6ST* and *IL10* have not been reported previously. IL-6 levels and actions, namely, IL-6 signaling, are dependent on the two receptors IL-6R and gp130. gp130, encoded by *IL6ST*, is a common receptor subunit that is shared with other receptors belonging to the IL-6 cytokine family. Different pathways of IL-6 signaling represent different types of cells that respond to IL-6; this could explain both the pro- and anti-inflammatory actions of IL-6 cytokine [35,36]. In infants that subsequently developed BPD, increased concentrations of IL-6 and soluble forms of IL-6R and gp130 were observed with different ratios in tracheal aspirate suggesting that altered IL-6 signaling may have a role in pulmonary inflammation [37]. Additionally, high cord blood levels of soluble gp130 at birth have been shown to predict subsequent development of BPD among very preterm infants [15], and the role of IL-10 in BPD has been investigated [13,38]. Thus, the potential interaction we observed in this study may be biologically relevant, but it requires further investigation.

The strengths of our study are the use of genetically relatively homogenous Finnish populations, the availability of multiple populations of European descent (i.e. we did not include individuals of clearly different ethnicities to avoid bias arising from population structure), and the strict phenotypic criteria among the participating centers. We analyzed common polymorphisms of several genes with potentially relevant roles in the pathogenesis of BPD. Possible limitations of this study are that the initial study population (northern Finnish) was relatively small and that the suggestive interaction was not observed in the other populations. Our northern Finnish population is likely more genetically homogenous than populations that originated in other parts of Finland [39] and compared to other populations of European origin [40]. This could have affected the different patterns of interaction between SNPs that we observed among the different populations in this study. An additional replication study is needed, with a larger population.

## Conclusions

None of the polymorphisms within the *IL6*, *IL6R*, *IL6ST*, *IL10*, *TNF*, and *NR3C1* genes were associated with BPD susceptibility, nor were they associated with the measured biomarkers. We observed a modest evidence of epistasis, i.e., a potential SNP–SNP interaction that could be associated with the risk of BPD. To our knowledge, epistasis has not been previously addressed in genetic studies of BPD. We propose that further studies of gene–gene interactions in the etiology of BPD are needed.

## Additional file

Additional file 1:
**Additional data for “A study of genes encoding cytokines (** ***IL6*** **,** ***IL10*** **,** ***TNF*** **),** **cytokine receptors (** ***IL6R*** **,** ***IL6ST*** **), and glucocorticoid receptor (** ***NR3C1*** **) and susceptibility to bronchopulmonary dysplasia” by Huusko JM, Karjalainen MK, Mahlman M, Haataja R, Kari MA, Andersson S, Toldi G, Tammela O, Rämet M, Lavoie PM, and Hallman M.**

## Abbreviations

BPD: Bronchopulmonary dysplasia
FU: Fluorescence unit
GA: Gestational age
HWE: Hardy–Weinberg equilibrium
IUGR: Intrauterine growth restriction
LD: Linkage disequilibrium
MAF: Minor allele frequency
OR: Odds ratio
PMA: Postmenstrual age
SNP: Single nucleotide polymorphism
tSNP: Tagging SNP
