# Supplementary material for: A study of genes encoding cytokines (IL6, IL10, TNF), cytokine receptors (IL6R, IL6ST), and glucocorticoid receptor (NR3C1) and susceptibility to bronchopulmonary dysplasia
Source: BMC Med Genet. 2014 Nov 1;15:120. doi: 10.1186/s12881-014-0120-7 (PMC4258941; doi:10.1186/s12881-014-0120-7)

Additional data for “A study of genes encoding cytokines (*IL6*, *IL10*, *TNF*), cytokine receptors (*IL6R*, *IL6ST*), and glucocorticoid receptor (*NR3C1*) and susceptibility to bronchopulmonary dysplasia” by Huusko JM, Karjalainen MK, Mahlman M, Haataja R, Kari MA, Andersson S, Toldi G, Tammela O, Rämet M, Lavoie PM, and Hallman M

Figure 1. Representative pairwise D prime linkage disequilibrium (LD) plots of the genotyped polymorphisms of (A) *IL6R* and *IL10* genes in chromosome 1, (B) *IL6* in chromosome 7, (C) *IL6ST* and *NR3C1* in chromosome 5, and (D) *TNF* in chromosome 6. LD plots are shown for the initial northern Finnish population. Names and relative positions of the polymorphisms are shown on the top. Numbers in the squares are pairwise D′ values; the darker the square, the higher the LD. Relatively strong LD was present within the genes; therefore, redundant SNPs were excluded from the epistasis analysis. Only one SNP from each haploblock was included, and SNPs showing deviation from HWE were excluded, resulting in 22 SNPs that were included in the analysis.

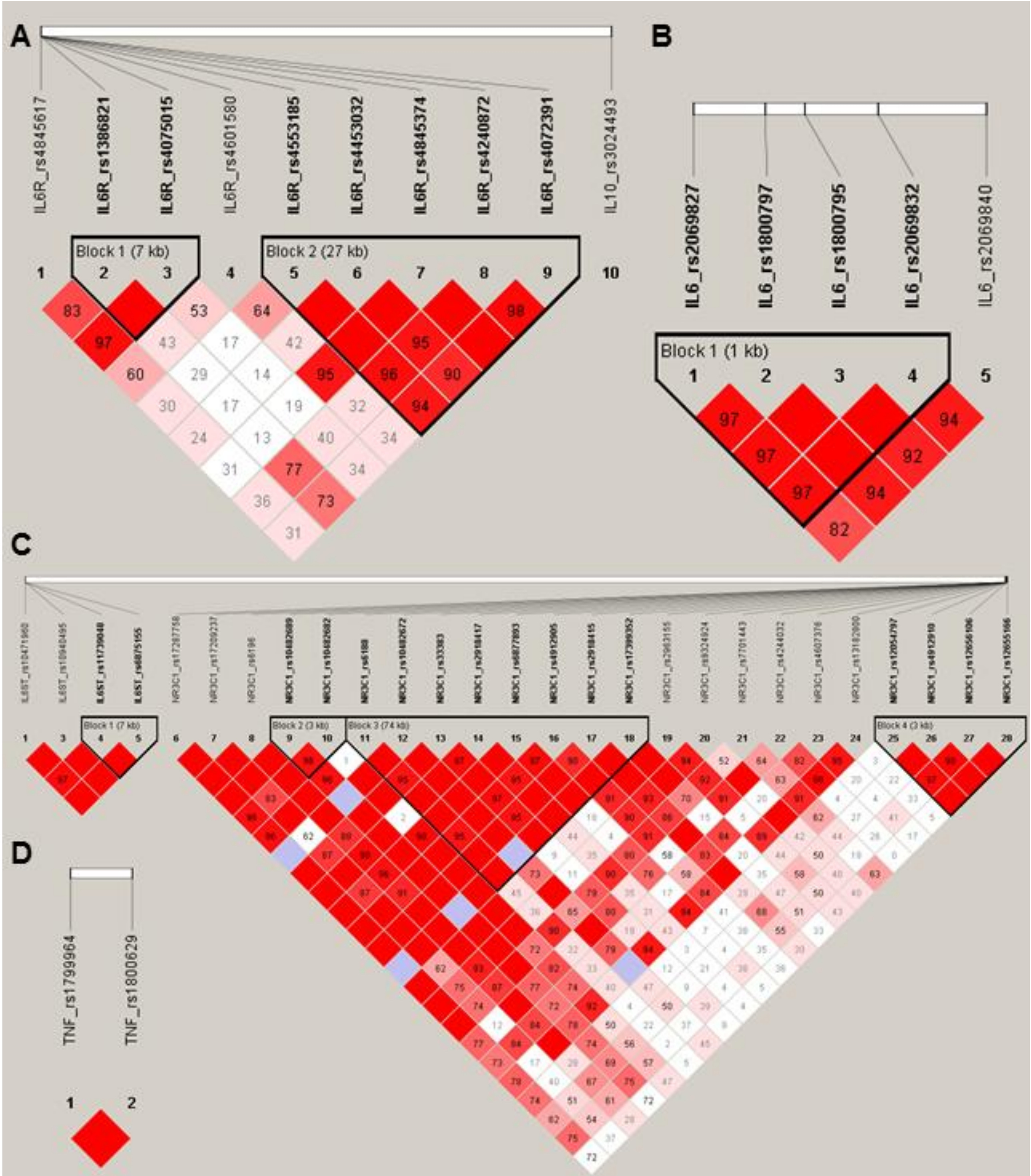

Supplement: Additional file 1: — Additional data for “A study of genes encoding cytokines ( IL6 , IL10 , TNF ), cytokine receptors ( IL6R , IL6ST ), and glucocorticoid receptor ( NR3C1 ) and susceptibility to bronchopulmonary dysplasia” by Huusko JM, Karjalainen MK, Mahlman M, Haataja R, Kari MA, Andersson S, Toldi G, Tammela O, Rämet M, Lavoie PM, and Hallman M. [file 12881_2014_120_MOESM1_ESM.pdf]
